# Potential hominin affinities of *Graecopithecus* from the Late Miocene of Europe

**DOI:** 10.1371/journal.pone.0177127

**Published:** 2017-05-22

**Authors:** Jochen Fuss, Nikolai Spassov, David R. Begun, Madelaine Böhme

**Affiliations:** 1 Department of Geoscience, Eberhard-Karls-University Tübingen, Sigwartstr. 10, Tübingen, Germany; 2 Senckenberg Centre for Human Evolution and Palaeoenvironment (HEP). Sigwartstr. 10, Tübingen, Germany; 3 National Museum of Natural History, Bulgarian Academy of Sciences, 1 Blvd Tzar Osvoboditel, Sofia, Bulgaria; 4 Department of Anthropology, University of Toronto, Toronto, Ontario, Canada; Université de Poitiers, FRANCE

## Abstract

The split of our own clade from the Panini is undocumented in the fossil record. To fill this gap we investigated the dentognathic morphology of *Graecopithecus freybergi* from Pyrgos Vassilissis (Greece) and cf. *Graecopithecus* sp. from Azmaka (Bulgaria), using new μCT and 3D reconstructions of the two known specimens. Pyrgos Vassilissis and Azmaka are currently dated to the early Messinian at 7.175 Ma and 7.24 Ma. Mainly based on its external preservation and the previously vague dating, *Graecopithecus* is often referred to as nomen dubium. The examination of its previously unknown dental root and pulp canal morphology confirms the taxonomic distinction from the significantly older northern Greek hominine *Ouranopithecus*. Furthermore, it shows features that point to a possible phylogenetic affinity with hominins. *G*. *freybergi* uniquely shares p4 partial root fusion and a possible canine root reduction with this tribe and therefore, provides intriguing evidence of what could be the oldest known hominin.

## Introduction

Within the intensively studied field of early hominin evolution, a crucial question is the split of our own clade from the Panini. Over the last decades the fossil record of potential early hominins increased with taxa such as *Ardipithecus*, *Orrorin* and *Sahelanthropus* [1–3]. Recent molecular data propose a divergence time of *Pan* and *Homo* between 5 and 10 Ma [4] and Langergraber *et al*. [5] propose an age of at least 7–8 Ma. These estimations largely coincide with the evidence obtained from the fossil record across Africa and Eurasia [6, 7].

In the present study, we define ‘hominoid’ as ‘apes’; ‘hominid’ as ‘great apes and humans’; ‘hominine’ as ‘African apes and humans’; and ‘hominin’ as ‘humans and their non-ape ancestors’. Currently, the fossil record reveals three Miocene candidates with potential hominin affinity. *Ardipithecus kadabba* is dated to between 5.2 and 5.8 Ma. It is more primitive than *Ardipithecus ramidus* and may not belong to the same genus [8], but it does show hominin affinities such as evidence of bipedalism and canine reduction [9, 10]. *Orrorin tugenensis* is dated to ~5.8–6.0 Ma and shows an upright posture [2, 11]. *Sahelanthropus tchadensis* is dated to ~6–7 Ma [3, 12] and provides several derived cranial and dental features that suggest hominin affinity. Lebatard et al. [13] propose an age of 7.2–6.8 Ma for *Sahelanthropus*. We do not consider this age determination to be reliable given the circumstances of the provenance of the skull [14] and the relatively low accuracy of the method [15].

The overwhelming effort to reconstruct hominin origins have been focused on the African continent. However, ancestral lineages remain largely unknown [16]. A crucial problem in identifying ancestral lineages is the prevalence of homoplasy and the relative lack of derived morphological features that reduces the phylogenetic resolution around lineage divergence [17, 18]. Root morphology might be a potential feature, which is less affected by homoplasy. Studies on fossil hominids, extant great apes and humans indicate that the premolar root number is not primarily linked to a functional adaptation, and is interpreted to represent a genetic polymorphism [19, 20]. Hence, homoplasy is only a minor consideration for the traits of premolar root numbers, which therefore may provide a useful phylogenetic signal. Nevertheless, some relations of root and crown morphology indicate overlaying masticatory adaptations that may attenuate the phylogenetic signal [21, 22].

Of special importance for hominin evolution is the lower fourth premolar (p4), as its morphology seems to be diagnostic for the hominin lineage. Taxonomic attempts have been made concerning its crown morphometry [23–25] and especially its root configuration [26, 27], which turns out to be a powerful tool for early hominin phylogeny [28]. Several morphological traits of putative early hominin p4s (*Sahelanthropus*, *Ar*. *kadabba*, *Ar*. *ramidus*) point to a reduced configuration. A two-rooted, but narrow state is documented in *Sahelanthropus* [28, 29]. A Tomes’ root is present in *Ardipithecus kaddaba* and a single-rooted p4 is characteristic for *Ardipithecus ramidus* [1, 30, 31] and *Homo*. The plesiomorphic p4 root configuration shown by extant great apes, basal hominids like *Proconsul* and Miocene hominines (*Ouranopithecus*) differs significantly, showing two or three clearly diverging roots and four pulp canals [28, 32]. The p4 root number in australopithecines (*Au*. *anamensis*, *Au*. *afarensis*, *Au*. *africanus*; [33–37]) is highly variable, from a Tomes’ root up to a three-rooted condition [26]. Another p4 root morphology, which has two roots that are fused on their basal buccal part, is recently described for some specimens of *P*. *robustus*, *Au*. *africanus* and australopithecines from Woranso-Mille [25, 36].

**Fig 1:**
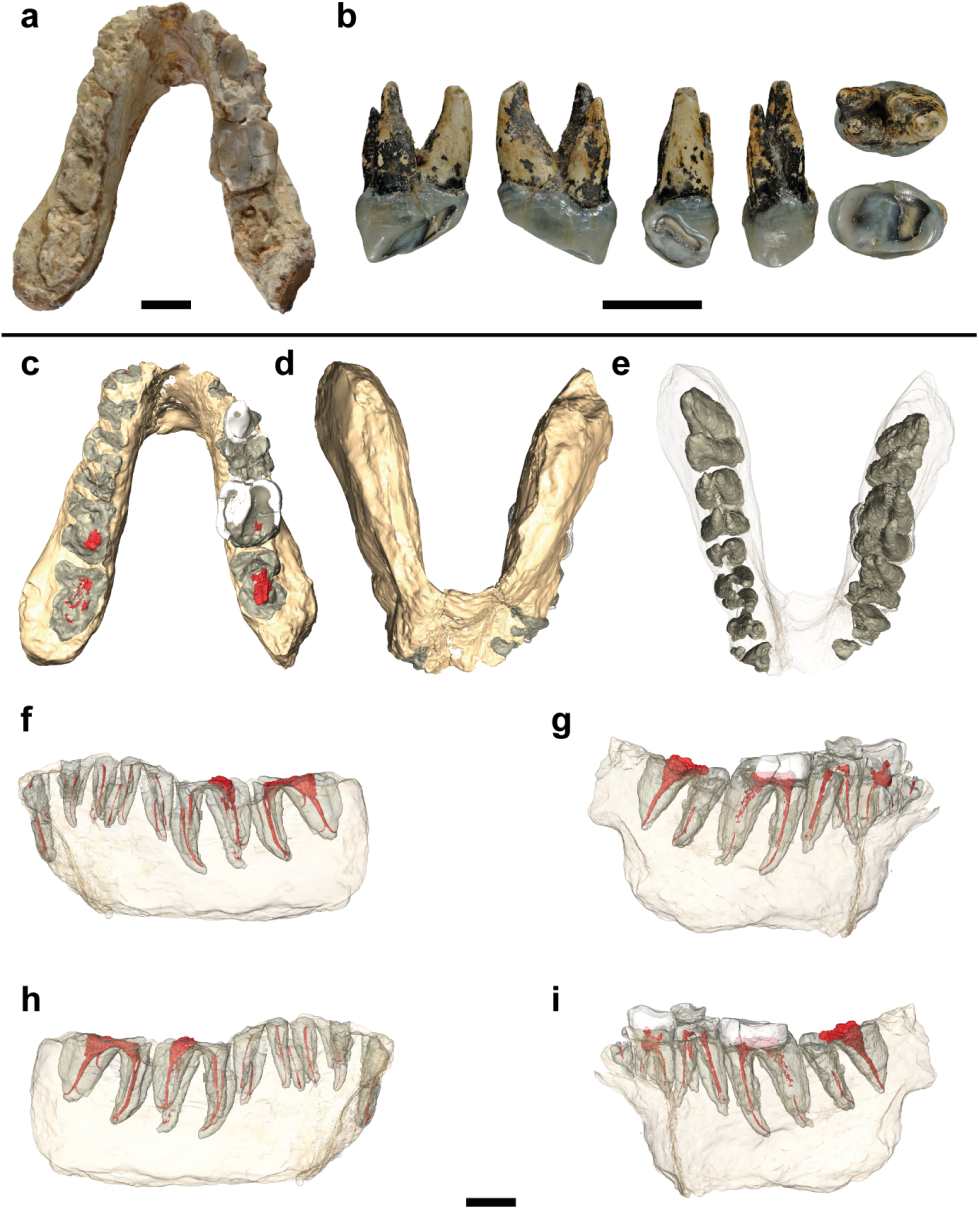
Studied specimens and virtual reconstructions of the holotype of *Graecopithecus*. **a**, Type mandible of *G*. *freybergi* from Pyrgos, Greece. **b**, RIM 438/387 –Left P4 of cf. *Graecopithecus* sp. from Azmaka, Bulgaria. From left to right: distal, mesial, lingual, buccal, occlusal and apical. **c-i**, μCT based 3D reconstructions of the type mandible showing the partially preserved roots and pulp canals from c-m3 and the crowns of right p4-m2. Further images with a magnification of the virtually isolated teeth and pulp canals are provided in S1 Fig. **c**, Occlusal view. **d-e**, Apical view. **f**, Buccal view of the left hemimandible. **g**, Buccal view of the right hemimandible. **h**, Lingual view of the left hemimandible. **i**, Lingual view of the right hemimandible. Scale bars, 10 mm.

In this study, we propose based on root morphology a new possible candidate for the hominin clade, *Graecopithecus freybergi* from Europe. *Graecopithecus* is known from a single mandible from Pyrgos Vassilissis Amalia (Athens, Greece) [38] and possibly from an isolated upper fourth premolar (P4) from Azmaka in Bulgaria [39] (Fig 1A and 1B). A new age model for the localities Pyrgos Vassilissis and Azmaka, as well as the investigations on the fauna of these localities [40] confirms that European hominids thrived in the early Messinian (Late Miocene, 7.25–6 Ma) and therefore existed in Europe ~ 1.5 Ma later than previously thought [39]. This, and recent discoveries from Çorakyerler (Turkey), and Maragheh (Iran) demonstrate the persistence of Miocene hominids into the Turolian (~8 Ma) in Europe, the eastern Mediterranean, and Western Asia [41, 42].

The type mandible of *G*. *freybergi* was found in 1944 by von Freyberg, who mistook it for the cercopithecid *Mesopithecus* [43]. In the first description by von Koenigswald [38] the mandible was identified as a hominid. Some authors have concluded, based on external morphology and in particular the apparently thick enamel and large molars, that another hominid from Greece, *Ouranopithecus* (9.6–8.7 Ma [44]), could not be distinguished from *Graecopithecus*, thus synonymizing the former with the latter [45]. Other authors have consistently maintained a genus level distinction between *Ouranopithecus* (northern Greece) and *Graecopithecus* (southern Greece), based on the argument that the Pyrgos specimen is insufficiently well preserved to diagnose a taxon (nomen dubium) or based on anatomical arguments [6, 44, 46].

Here, we provide a detailed description of the Pyrgos and Azmaka specimens by using μCT based analyses and 3D visualisations. For the first time, their internal structures are examined in order to reveal previously unknown characters in root and pulp canal morphology. Additionally, previously described features are re-assessed and a new diagnosis of *G*. *freybergi* is given. Thereby, we address the taxonomic validity of *G*. *freybergi* and further, raise the possibility of a hominin affinity.

## Material and methods

The studied material comprises the type specimen of *Graecopithecus freybergi* from Pyrgos Vassilissis Amalia (Athens, Greece)—a mandible with partially damaged permanent dentition (c-m3, Fig 1A) and RIM 438/387—an upper fourth premolar of cf. *Graecopithecus* sp. from Azmaka (near Chirpan, Bulgaria; Fig 1B). The fossil sites are dated to the early Messinian at 7.175 Ma (Pyrgos Vassilissis) and 7.24 Ma (Azmaka; AZM 4b) [40].

Comparative data of fossil and extant great apes and humans were obtained from casts (*O*. *macedoniensis*/RPl-54) and the literature. Selecting criteria for the comparative taxa has been the availability of appropriate data from literature. Accordingly, the literature data needs to describe the same anatomical structures that are preserved in *G*. *freybergi* (e.g. dental root morphology, number and length, corpus dimensions, etc.). Further, attention was paid to the comparability of measurements, which is specifically discussed in the methodical section. Thus, the set of comparative taxa may vary between the investigated characters.

The type mandible of *G*. *freybergi* was found in 1944 during construction of a German bunker [43]. Situated in the urban area of Athens, the fossil site is overbuilt and thus not accessible anymore. The mandible and further vertebrate fossils were deposited in reddish fine sediments of Late Miocene age.

A first preparation of the mandible was done by von Koenigswald [38]. For further studies [45] it was brought to the Natural History Museum in London, where it has been completely cleaned of the surrounding matrix. The damaged external face of the symphysis has been treated with resin, which has stabilized the preserved internal face of the symphysis.

### μCT and virtual reconstruction

Both halves of the mandible and the Azmaka tooth were separately scanned with the GE Phoenix v|tome|x s μCT scanner at the Institute for Archaeological Sciences (INA, University of Tübingen, Germany). The Pyrgos and Azmaka scans have a resolution of 29.48 μm and 21.44 μm, respectively. The specimens were scanned at 170/150kv and 170/140 μA. No beam hardening artefacts were observed. The μCT slice data were converted into 3D volumes using Avizo 8.0 software (FEI Visualization Sciences Group). The fossil material was virtually isolated from the background, the adhesives and rock particles. Further, the density contrast of bone, dentine, enamel and filled cavities was used to segment specific anatomical elements of the mandible (mandibular bone, dental crowns, roots and pulp cavities/canals). The segmentation was complicated by the low-density contrast of the Pyrgos scan, thus implying both manual and semiautomatic segmentation of each anatomical element slice by slice (4578 slices in total). This was done with a combination of surface determination, region growing and masking tools. For further processing in Geomagic Wrap 3.0 software (3D Systems Corporation), smaller datasets were required. Therefore, the surfaces of the reconstructed elements have been simplified in Avizo. The extracted STL-files were transferred to Geomagic, where both halves of the mandible were digitally repositioned and finally smoothened for presentation purposes (Fig 1C).

### Mandibular measurements

Mandibular measurements were taken on the type of *G*. *freybergi* and a cast of the type of *O*. *macedoniensis* (RPL-54). Further comparative data of *O*. *macedoniensis* (NKT-21, RPL-90, RPL-80, RPL-56, RPL-75, RPL-89, RPL-94) was obtained from the literature [47, 48]. Additional mandibular measurements from literature come from the taxa *Ankarapithecus meteai* [49], *Sivapithecus sivalensis* and *S*. *punjabicus* [45], *Nakalipithecus nakayamai* [50], *Australopithecus anamensis* [51], *Au*. *deyiremeda* [52], *Au*. *afarensis*, *Au*. *africanus*, early *Homo*, *Paranthropus robustus* and *P*. *boisei* [52]). The measurements on *G*. *freybergi* were made using the Avizo 3D measuring tool directly on the μCT-slices or the un-smoothened 3D reconstruction. The cast of RPL-54 was measured with a calliper gauge (accuracy = 0.02 mm). Unless otherwise stated, all values are given in millimetres and rounded to one decimal.

For the mandibular dimensions, the corpus height (H) and breadth (B) were measured at the positions between and below each tooth. The measurements were performed on the μCT-slices oriented perpendicular to the alveolar plane. The measurement of the corpus breadth accords with a measurement with a calliper that is aligned on the lingual corpus side. The corpus height was measured lingually, perpendicular to the breadth measurement, as shown in S2 Fig. The mandibular robusticity index (RI) was calculated as the ratio W/H. Further, the μCT-sections (S2 Fig) were taken in each position to ensure the reliability of the corpus dimensions in *G*. *freybergi*. The sections show that the mandible is crushed ventrally and the outer cortex is partially missing. This mainly concerns the right hemimandible. Therefore, the breadth-height measurements were restricted to the better-preserved left corpus. Particularly, in the position of m2/m3 to m3 the outer cortex and the trabecular bone are largely preserved. Hence, a reliable breadth can be given here. A minimal estimation is given for the breadths at p3/p4 to m2. A small amount of damage on the lower rim is reconstructed as shown in S2 Fig. Accordingly, a minimal estimation is given for the corpus depth in the position from m2 to m3.

The mandibular symphysis preserves only parts of the internal (lingual) face. Therefore, its symphysal height and breadth are not measurable. To assess its limited morphology, three anatomical planes were constructed on a sagittal μCT-cross section: alveolar plane (AP), sublingual plane (SP) and plane of transvers tori (TP = bitangent of the upper and lower transvers tori). The angle of SP and TP with AP is measured, as well as the angle of SP with TP. Comparative symphysal cross-sections of *O*. *macedoniensis* (RPl-56, RPl 75, RPl-54) were obtained from literature [53].

The width of the dental arcade is measured on the repositioned 3D reconstruction of *G*. *freybergi* and the cast of RPl-54. The distances were taken lingually at the cervix of each tooth. The slight distortion of left and right hemimandible is considered here to be minor and thus, the un-corrected direct measurements are provided. Although the Pyrgos mandible is broken, the distance between both hemimandibles is determinable as the internal face of the symphysis is continuously preserved.

### Dental crown measurements

The tooth crown dimensions were measured with the 3D measuring tool of Avizo 8.0 on the un-smoothened virtual reconstruction of the Pyrgos specimen. The length (mesiodistal) and width (buccolingual) was measured for the preserved right m2 crown. In p4 only the mesiodistal length is measurable as parts of the buccal crown are broken. Tooth row lengths must be used with caution as the teeth of the Pyrgos specimen are severely crowded and show intense interstitial wear. Particularly, the m1 crown is strongly affected by interstitial wear and lateral crushing. In order to get an approximation of its original size, we applied the tooth area prediction following Evans et al. [54]. We used the estimation model developed for australopithecines and calculated the crown size derived from the known m2 dimensions. The application of this model to taxa other than intended by Evans *et al*. must be used with caution and needs a throughout investigation first. A first hint of its applicability for our purpose was tested with the well-preserved dentition in the type of *O*. *macedoniensis*. Comparative data for the crown dimensions in the m2 of *O*. *macedoniensis*, *O*. *turkae*, *N*. *nakayamai and A*. *meteai* [41, 47–50, 55] and the P4 of cf. *Graecopithecus* sp., *O*. *macedoniensis* and *O*. *turkae* [39, 41, 48, 56] were obtained from literature. Additional literature data of crown dimensions in the p4, m2 and P4 of other taxa (*S*. *tchadensis*, *O*. *tugenensis*, *Ar*. *kadabba*, *Ar*. *ramidus*, *A*. *afarensis*, *A*. *anamensis*, *P*. *troglodytes*) is obtained from [1–3, 9, 33, 57].

The enamel thickness was measured for the P4 from Azmaka and the right p4 and m2 of the Pyrgos specimen. The enamel of m1 was too fragmentary for quantification. Relative enamel thicknesses could not be applied, due to the intense dental wear. Hence, two dimensional measurements were taken following Suwa & Kono [58]. Abbreviations are adopted from [58, 59]:

MCS: mesial cusp section. Section through the dentine horn tips of the metaconid and the protoconid.

l: radial enamel thickness on the lingual side of the metaconid.

k: radial enamel thickness on the buccal side of the protoconid.

The teeth were virtually sectioned in Avizo through the mesial dentine horn tips (MCS) from buccal to lingual. The generated CT-sections were directly used for the two dimensional linear measurements. Due to the intense occlusal and interstitial wear, the enamel on the lateral sides provides the least altered thicknesses. Hence, we took the radial enamel thickness only on lingual (l) and buccal (k) side of each tooth. The buccal side of lower molars can further be altered if there is a Carabelli’s cusps in the opponent upper molar. Therefore, we measured the lingual side of the lower teeth and the buccal side of the upper teeth [60].

The μCT-based measurements were taken at a resolution of ~30μm and are given in millimetres, rounded down to the first decimal place. The published radial enamel thicknesses used for comparison [41, 58–60] are derived from differing methodologies. This mainly concerns earlier studies that used physically sectioned teeth. This method produces uncertainty that the MCS are not exactly positioned at the dentine horn tips. Martin [59] cut a mesial section through the tips of the enamel cusps, assuming that the dentine horn tips lie exactly underneath. However, this is not always the case. Grine [60] sectioned the teeth distal to the enamel cusps to ensure that the dentine horn tips remain. Afterwards, the cut surface of the mesial block was grounded down until the dentine horn tips are reached. The measurements were then derived from SEM-micrographs of the MCS. Today, radial thicknesses are measured by μCT with a resolution of 40 μm and 56μm [41, 58]. Accordingly, inter-observer errors between these studies can be expected. Considering these limitations, the present comparison of enamel thicknesses has the aim to show the large-scale differences (thin/medium/thick enameled) between taxa. The comparative samples consist of male and female specimens in unbalanced proportions, assuming no significant sexual dimorphism in molar absolute enamel thicknesses [61, 62]. In addition, the sex of fossil specimens is not always known, so a bias towards males or females cannot excluded. The specimens of *Homo sapiens* are from diverse archaeologically derived and recent populations [58–60].

### Root length

The measurement of the root length follows Moore et al. [63] and was performed with the 3D measuring tool in Avizo 8.0. The measurement is done linearly from the root apex to the point, where the pulp canal cuts the cervical plane. Thereby, the measurement largely follows the course of the pulp canal. We considered only the longest radical of each tooth (maximal root length). For *G*. *freybergi* these are the following positions: single root-apex of c, distobuccal root-apex of m1, mesiobuccal root-apex of p3, p4, m2 and m3.

Estimated corrections (S3 Fig): The root lengths of the left m2 and the right molars (m1-m3) are completely preserved and the maximal root lengths can directly be measured. The canine and premolars are only partially preserved. The right p4 lacks the apical root tips and the right p3 only preserves a fragment of the distal root. In the left hemimandible the upper parts of the roots of c-m2 are eroded, but the apical root tips are all preserved. Though this preservation does not allow a direct measurement of root length, an estimation of their final root lengths can be made. The corrected measurements on the canine and premolar roots can be derived from the apical root depths known from the left c-m3 and the right m1-m3. The cervical planes preserved in the right hemimandible provide the upper limit. As the mandibular corpus is slightly distorted, it is not possible to create a simple cervical plane across both halves. In order to bring them into the same vertical plane, the left hemimandible was mirrored and aligned to the right one via the software Geomagic Wrap 3.0. The positioning of both hemimandibles was done by aligning the left and right m1-m3 at their points of root bifurcation. Thereby, the left canine and premolar roots were transferred to the right side, where the cervical planes were largely preserved. The cervical planes were constructed through the cervices of the right m2-p4 and were extended to the position of p3 and c. Hence, the upper and lower ends of the p4, p3 and canine roots are defined by the cervical plane of the right hemimandible and the apical root tips of the left hemimandible.

Comparative data: The comparative root lengths data of extant hominids (*Pongo pygmaeus*, *Gorilla gorilla*, *Pan troglodytes* and *Homo sapiens*) are from Abbott [64]. The comparative fossil taxa include *S*. *tchadensis* [28], *Ar*. *ramidus* [31], *Au*. *anamensis* and *Au*. *afarensis* [65]. For extant hominids, the minimum, maximum, mean and standard deviation is given for the root lengths of males and females. The fossil hominids are sex-pooled or not assigned to sex. Minimum, maximum, mean and sample size (n) are given.

Some comparative studies used slightly different methods of root length measurements.

Abbott [64] derived root lengths from 2D radiographs and measured an *actual root height* of each root of a tooth. The *actual root height* means the apico-cervical distance along the root axes and thus, largely resembles our measurements. For comparison, we choose the same root positions that we measured on *G*. *freybergi*: single canine root, distal root in m1, mesial root in p3, p4, m2 and m3. Similar to our root length measurements on *G*. *freybergi*, the comparative data of *S*. *tchadensis* are maximum root lengths that are measured on 3D reconstructions [28]. In *Ar*. *ramidus*, *Au*. *anamensis* and *Au*. *afarensis* the canine lengths used here were measured apico-cervically on original specimens and casts [31, 65].

### Root morphology

The dental root configuration follows the formula given by Emonet [32]. Thereby, the number and position of the roots and pulp canals are described for each tooth position:

χαM+үβD (for multi-rooted teeth) and 1_1_ (for single-rooted teeth with one pulp canal)

χ = mesial root number; ү = distal root number; α = number of mesial pulp canals; β = number of distal pulp canals; M = mesial; D = distal.

There have been several attempts to define the degree of bifurcation and the number of roots [22, 26, 27, 32, 66]. As our comparative data for root numbers largely comes from [32] and [28] we follow their definitions: Two free roots are counted if there is no fusion of dentine for more than one third of the total root length and both radicals have a distinct apex. If a lingual radical is connected to a buccal radical by a thin blade and both radicals are visible for more than half of its total length they are counted as two separate roots. For a better comparability to other studies, we provide figures of the root and pulp morphologies of each tooth of the Pyrgos and Azmaka specimen (S1 Fig).

## Description of the specimens

The Pyrgos specimen consists of a mandible with partially damaged dentition (c-m3). It belongs to an adult individual as indicated by the fully formed permanent dentition and the closed root apices. The tooth crowns of the right p4-m2 are partially preserved and the dental roots of the right p3-m3 and left c-m3 are largely preserved. The anterior mandibular body is snapped in two, separating both corpora, but the break is clean and the specimen is easily reassembled (Fig 1A, 1C and 1D). Both corpora show slight distortion and some damage, especially on the right side.

**Fig 2:**
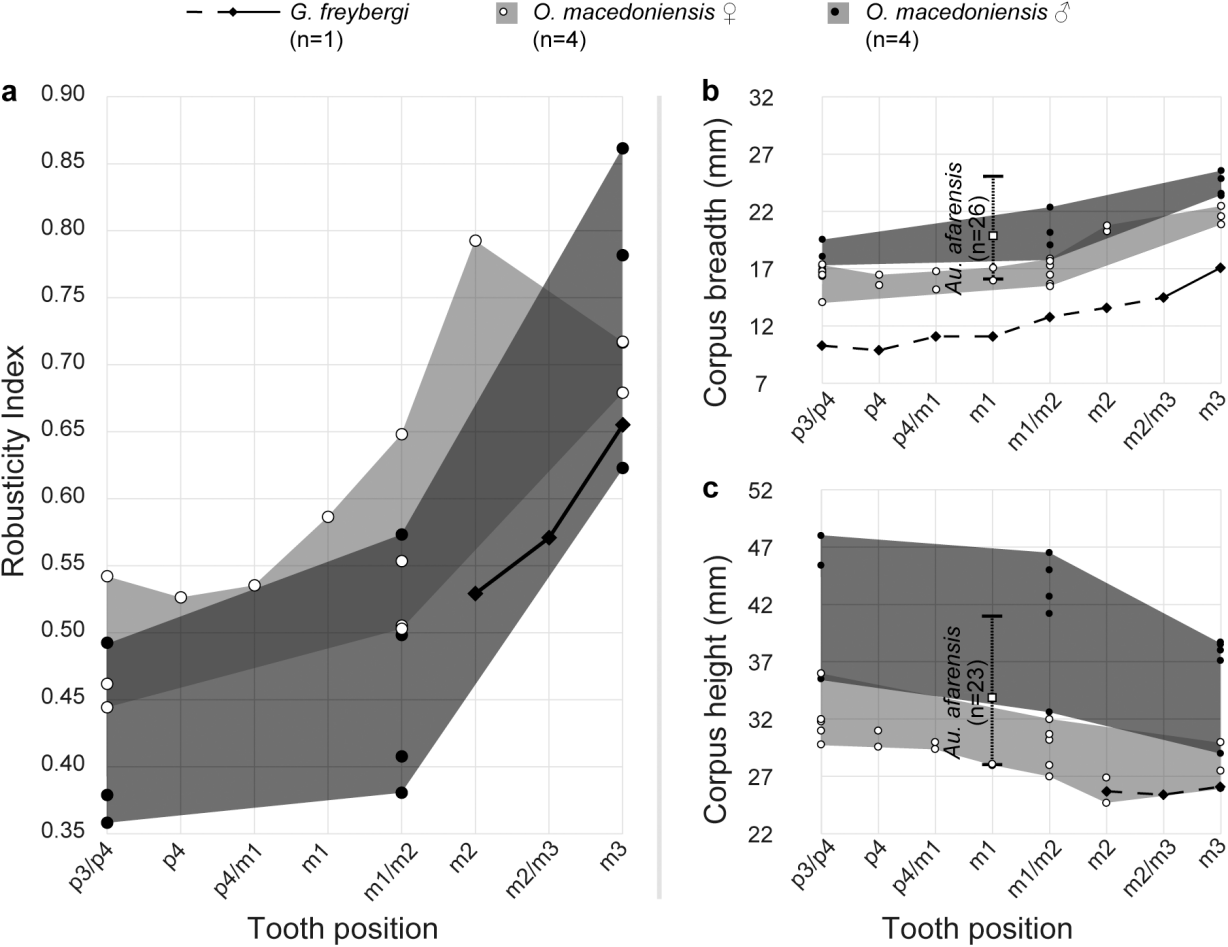
Robusticity and dimensions of the mandibular corpus in *G*. *freybergi* and *O*. *macedoniensis*. **a**, Mandibular robusticity index (RI = corpus breadth/height) in different tooth positions of *G*. *freybergi* compared to female and male *O*. *macedoniensis* (RPL-54, NKT-21, RPL-90, RPL-80, RPL-56, RPL-75, RPL-89, RPL-94; [47, 48] and this study). **b**, Corpus breadth and **c**, Corpus height in different tooth positions of *G*. *freybergi*, *O*. *macedoniensis* (RPL-54, NKT-21, RPL-90, RPL-80, RPL-56, RPL-75, RPL-89, RPL-94; [47, 48] and this study) and *Au*. *afarensis* [52]. In *G*. *freybergi*, the mandibular corpus is laterally crushed and is close to the real breadth only posterior to the left m2. Minimum estimations are indicated with dashed line. See also S2 Fig and S1 Table.

**Fig 3:**
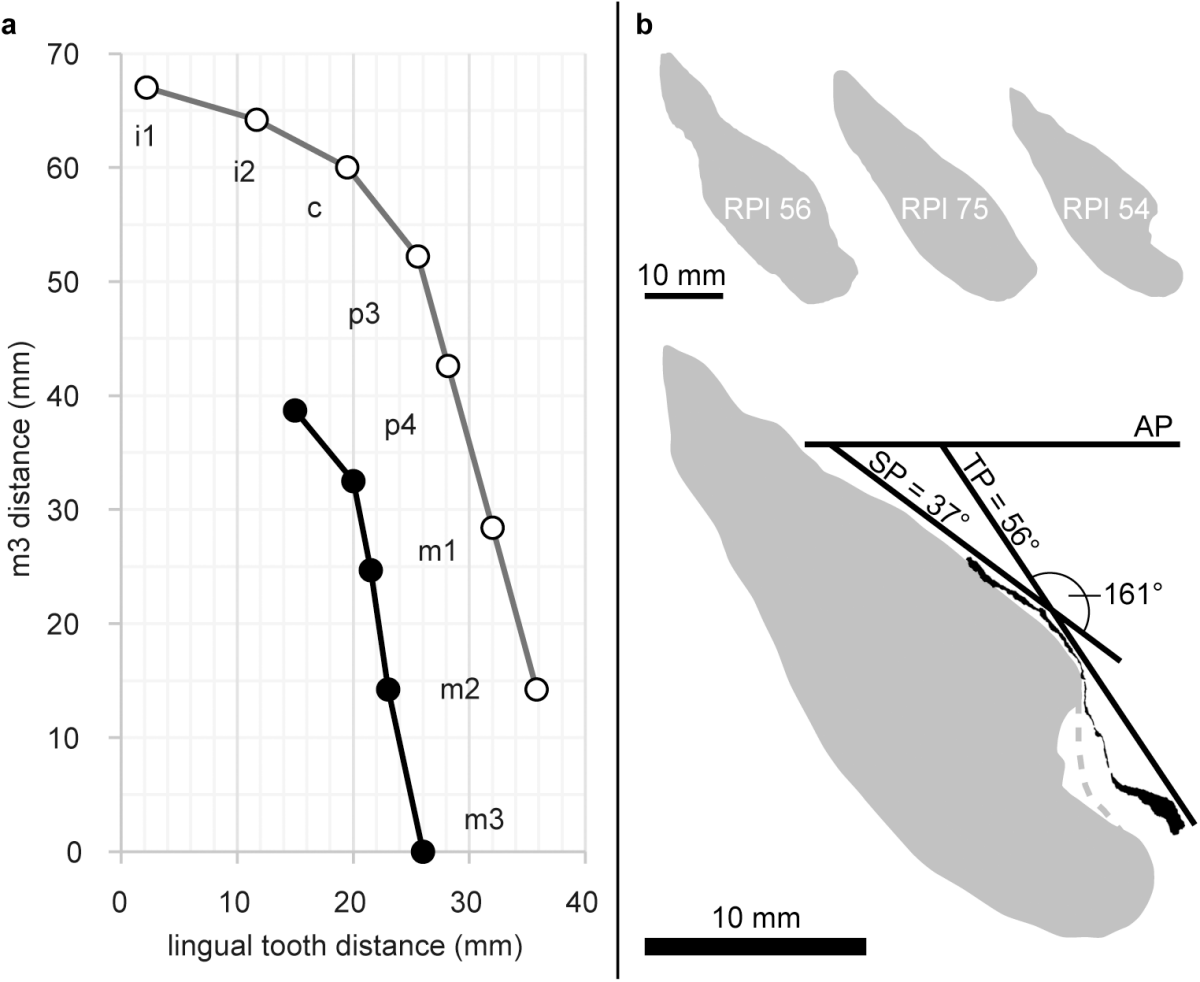
Morphometry of the mandibular corpus and symphysis in *G*. *freybergi* and *O*. *macedoniensis*. **a**, Bivariate plot of the mandibular tooth row of *G*. *freybergi* (black) and *O*. *macedoniensis* (RPl-54; grey) illustrating the differences in arcade width. The lingual distances between the left and right tooth row are plotted for each tooth position, if preserved. Measurement was done at the lingual sides of the dental cervices. The vertical axis shows the measuring position along the tooth row given as distance from m3. **b**, Top: Sagittal sections through mandibular symphyses of *O*. *macedoniensis* (RPl-56, RPl 75, RPl-54 [53]). Bottom: Sagittal section through the preserved veneer of the mandibular symphysis of *G*. *freybergi* (black) aligned to the symphysal sagittal section of *O*. *macedoniensis* [53] (RPl-54, grey; same scale). AP: Alveolar plane; TP: plane of transverse tori; SP: sublingual plane. The inclination of TP and SP, and the angle between both planes is given for *G*. *freybergi*.

The mandibular corpus is deep in cross section (tall relative to breadth.) Although the right mandibular corpus is crushed ventrally, a reliable breadth-height ratio is preserved on the left corpus from m2 to m3 (Fig 2, S2 Fig and S1 Table). The mental foramen preserved on the left corpus is positioned below the p4. It is situated ~6.0 mm from the mandibular base and ~22.5 mm from the alveolar margin. The dental arcade is narrow and divergent, with a distance of ~15 mm between the lingual sides of the p3 cervicesand ~26 mm at the m3s (Fig 3A and S2 Table).

The symphysis provides only limited information as it is mostly missing save a thin veneer (2–3 mm) of a portion of the lingual cortical bone surface (Fig 3B). The CT scans show that the anterior cortical and trabecular bone are missing and confirm that some cortical bone of the internal (lingual) surface is preserved. Hence, the lower part of the sublingual plane, the superior transverse torus (t.t.sup.) and the inferior transverse torus (t.t.inf.) are preserved (Fig 3B). The genioglossal fossa between both tori is shallow but clearly visible. The horizontal position of the t.t.sup. is at the level of the mid-p4, and the t.t.inf. is at the level between p4 and m1. The constructed bitangent of the t.t.sup. and t.t.inf. (plane of transverse tori; TP) forms an angle of 56° with the alveolar plane. The sublingual plane is oriented at an inclination of about 37°. The symphysal height and depth are not measurable.

**Fig 4:**
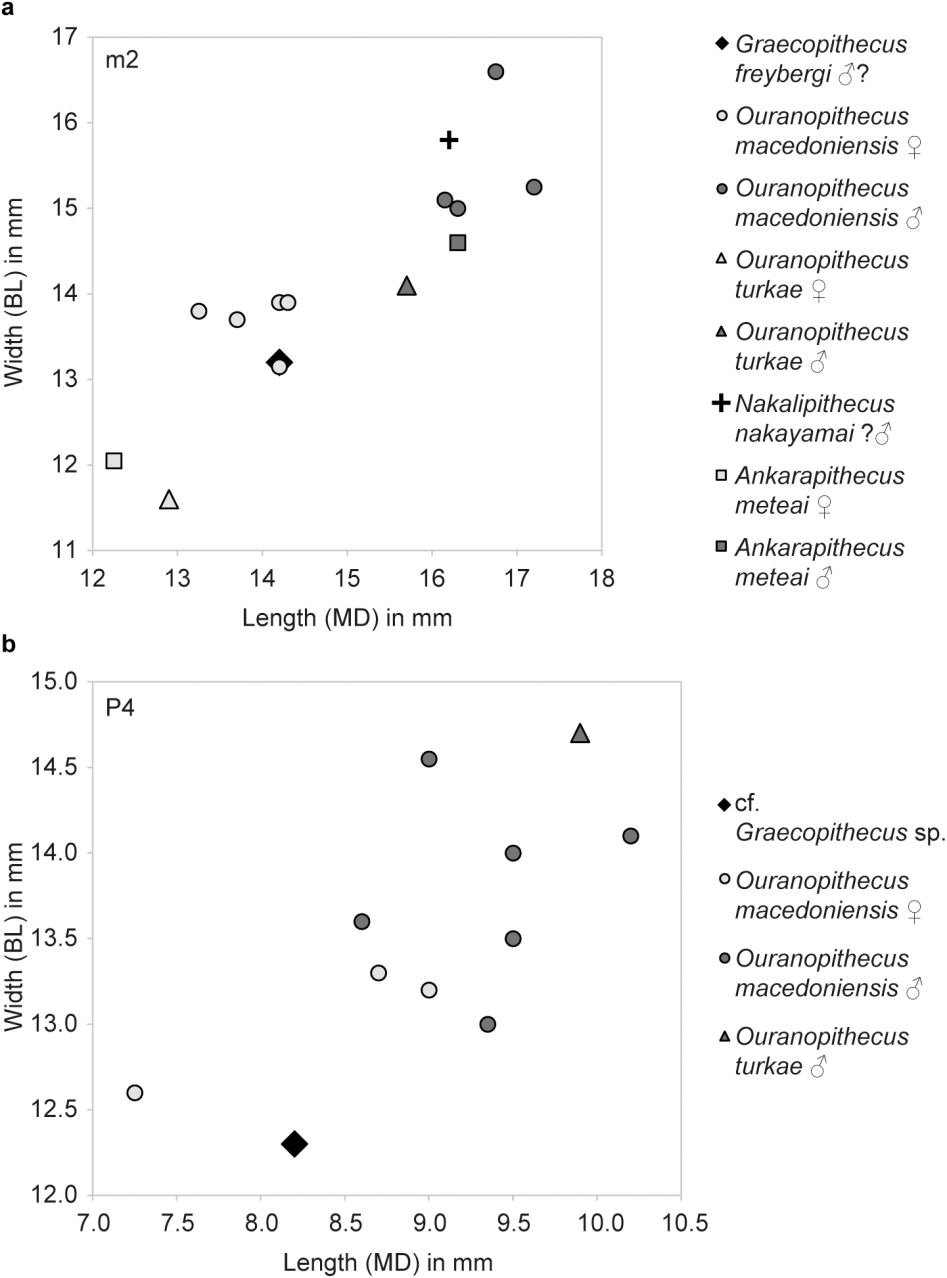
Dental crown dimensions of Late Miocene hominids. **a**, m2 crown dimensions of *G*. *freybergi*, *O*. *macedoniensis*, *O*. *turkae*, *N*. *nakayamai and A*. *meteai*. Comparative data: [41, 47–50, 55]. **b**, P4 crown dimensions of cf. *Graecopithecus* sp., *O*. *macedoniensis* and *O*. *turkae*. Comparative data: [39, 41, 48, 56].

**Fig 5:**
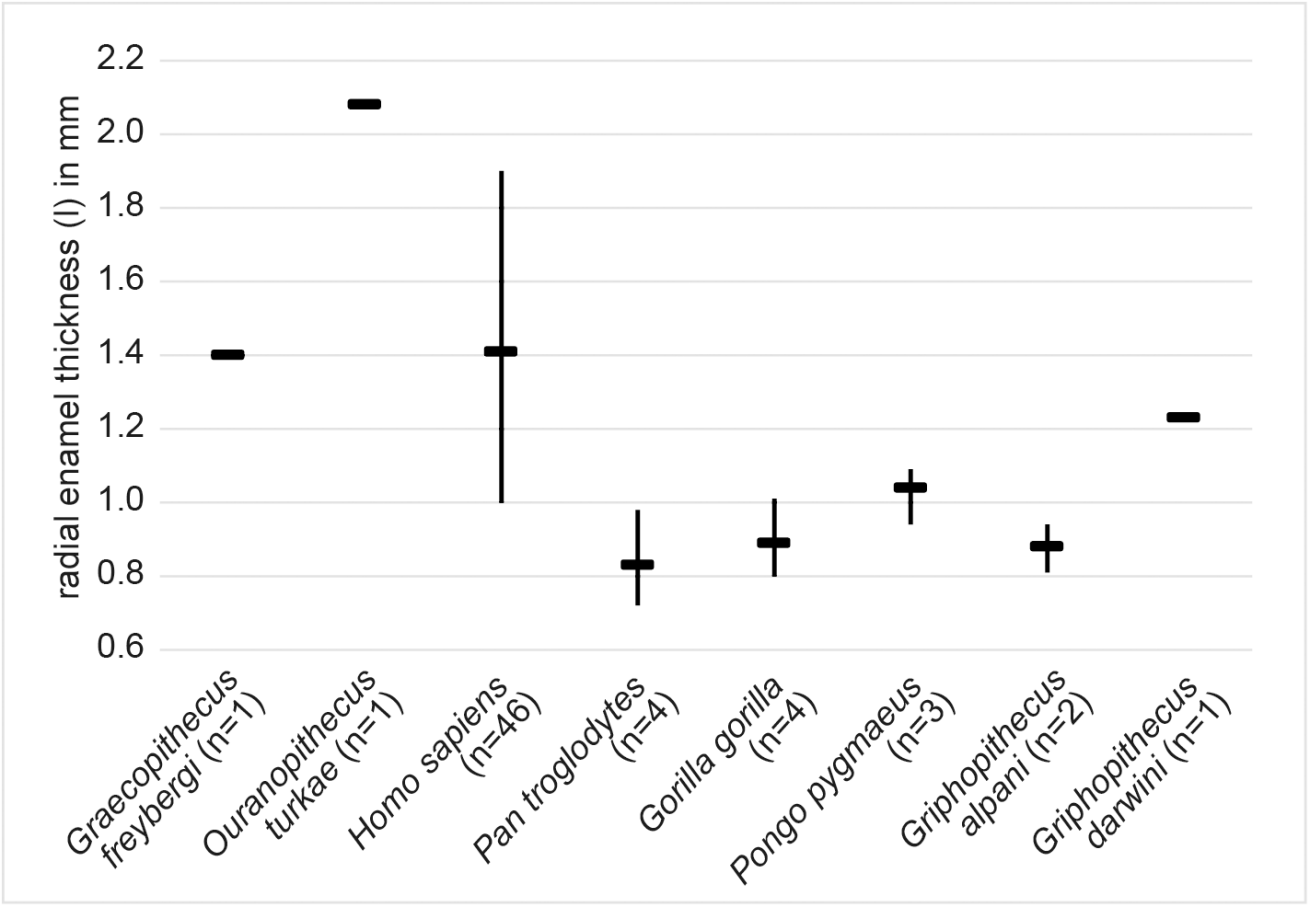
Radial enamel thickness in m2 of extant and extinct hominoids including *G*. *freybergi*. The lingual radial enamel thickness (l) in *G*. *freybergi* is measured on μCT slices at the lingual side of the metaconid, following [58]. Comparative data: [41, 58–60]. Horizontal line = mean; vertical line = range.

The partially preserved crowns of right p4 (its mesiobuccal face is missing), m1 and m2 show extreme occlusal and interstitial wear (Fig 1C). The p4 retains only a thin layer of occlusal enamel. Dentine is exposed on its buccodistal half and the metaconid (wear stage 5, after [67]). Although the occlusal surface is largely flattened, a mesio-distal step is clearly visible between the mesial cusps and the talonid. The occlusal enamel of the m1 and m2 is almost completely worn away, exposing large parts of the dentine. In m1 the conids are entirely worn away and only the outer rim of enamel remains (wear stage 7). In m2 (wear stage 5–6) the abrasion is focused on the buccal conids, where a deep hollow reaches the pulp chamber. The entoconid and metaconid are still visible, but expose their dentine horns. Due to the interstitial wear the mesial face in m1 is S-shaped and in m2 concave (Fig 1C). The distal half of m1 is obliterated with the interstitial wear reaching deep into the dentine. Martin & Andrews [45] calculated a crown length reduction of 32% for this tooth. This is consistent with the estimated loss of 30% in m1 tooth area, when we apply the tooth size prediction after Evans et al. [54]. Reliable crown measurements can only be taken from m2 (BL = 13.2 mm, MD≈14.2 mm; Fig 4) and p4 (MD = 9.1 mm). Based on the cervical root areas the tooth size is estimated to increase from m1 to m3 [45]. The m2 is often referred to as being slightly broader than the mandibular corpus at this level [38, 44–46], which is seen as a unique character of *G*. *freybergi*. However, this is partially an artefact of crushing, as the μCT-section reveals (S2 Fig). The better-preserved left corpus shows a breadth similar to that of m2, which is nevertheless unique among hominoids. Hence, the posterior dentition still shows a clear evidence of megadontia relative to corpus dimensions, but perhaps less dramatically than previously thought. The teeth are thickly enamelled, with a lingual radial enamel thickness of 1.40 mm for m2 and 1.50 mm for p4 (Fig 5 and S5 Table). The m1 radial enamel thickness is not measurable. The pulp chambers of the molars (right m1 and m2; S1C Fig) are vertically narrow. Their upper surface is flat as their pulp horns are inconspicuous or lacking. The CT-scans reveal an accumulation of dentine in large parts of the pulp chamber and pulp horns. Dentine layers of less density may trace the original pulp chamber. Thus, an accretion of secondary dentine can be assumed, particularly on the roof and the horns of the pulp chambers.

The maximal root lengths (longest root of a tooth, measured on 3D) of the molars are (left/right) m1 >13.5/ = 14.5 mm; m2 >16.9/ = 17.6 mm; m3 = 15.6/16.9 mm. The left canine root (>16.1 mm) is partially preserved, but its upper mesial part is missing. However, it is possible to estimate its maximal length to the cervical plane (c ≈25.5 mm; S3 Fig).

RIM 438/387 –the left P4 from Azmaka [39] has an intensively worn crown and three well preserved roots (Fig 1B). The crown is mesio-distally narrow with rounded rectangular occlusal outline (MD = 8.2 mm; BL = 12.3 mm). The enamel is thick with a buccal radial thickness of k = 1.55 mm. The occlusal wear facet is mesio-labially inclined and exposes large parts of the lingual dentine (wear stage 4; after [67]), but only the tip of the buccal dentine horn (wear stage 2). The distal crown surface shows a distinct interstitial wear facet. The P4 has a maximal root length of 12.0 mm; its roots are mesio-distally compressed. The buccal roots are close to each other and are fused in the upper 3 mm. Each radical features a separate pulp canal (S1A Fig). The pulp chamber is tall with a distinct buccal pulp horn.

## Comparison and taxonomic validity

*G*. *freybergi* is only known from one mandible and possibly the tooth from Azmaka (Fig 1A and 1B). This compares with a relatively large number of *Ouranopithecus* specimens. *Ouranopithecus* has been synonymised with *Graecopithecus* by some [45]. Others emphasize the dentognathic differences between both taxa, but regard the Pyrgos specimen as largely uninformative due to its poor surface preservation and vague dating [44]. The new data provided here support previous conclusions that *Ouranopithecus* and *Graecopithecus* differ in significant numbers of characters more than adequate to recognize two different taxa with probable generic differences [41]. Beside shared characters between *G*. *freybergi* and *O*. *macedoniensis* (thick enamel [44, 68, 69], m2 crown dimension, symphyseal shape; Figs 3 and 4), both taxa differ in the dental arch, which is shorter and narrower in *G*. *freybergi* (Fig 3A). The width (BL) and length (MD) of the m2 crown is within the range of female *O*. *macedoniensis* (Fig 4 and S3 Table), but it is broader relative to the mandibular robusticity. The BL width of m2 approximates the breadth of the mandibular corpus at this position. Hence, the mandible of *G*. *freybergi* is very gracile compared to *O*. *macedoniensis* and other Miocene and Pliocene hominids (Fig 2 and S1 Table), as already suggested by von Koenigswald [38] and Martin & Andrews [45]. Generally, the mandibular corpus breadth in hominids show only minor sex differences, but is of taxonomic significance [70–72]. The breadth of female and male *O*. *macedonienis* mandibles are closer to one another than either is to *G*. *freybergi* (Fig 2B). Thus, the considerable lower breadth in *G*. *freybergi* strongly suggests a taxonomic difference.

In contrast, the mandibular robusticity is significant for sex discrimination in hominids [35, 70, 71]. Male *O*. *macedoniensis* are less robust (taller relative to breadth) than females. The mandibular height of *G*. *freybergi* overlaps with the height of female *O*. *macedoniensis*, but its robusticity is in the lower range of the gracile males (Fig 2A). Assuming a similar pattern of sexual dimorphism with robust mandibles in females and gracile mandibles in males, the very gracile mandible of *G*. *freybergi* relative to its m2 size and compared to *O*. *macedoniensis* and other Miocene and Pliocene hominids (S1 Table), suggests that the *Graecopithecus* type mandible may belong to a male individual.

*G*. *freybergi* and *O*. *macedoniensis* differ in the number of their dental roots and/or pulp canals (Table 1) showing a reduced configuration in *G*. *freybergi*. Further, the buccal fusion of the p4 roots differs from the separated roots in *O*. *macedoniensis* and other Late Miocene hominids (e.g. *O*. *turkae*; see figure 2 in [41]), but approximates the root form recently described in australopithecine specimens from Woranso-Mille, in *Au*. *africanus* and in *P*. *robustus* [25, 36]. Much variability is known for the root number and morphology within and among australopithecine species, from a Tomes’ root to a three-rooted morphology (e.g. [26, 36]). However, within the fossil record the p4 root fusion is a feature that appears exclusively in hominins. 12% of *P*. *robustus* (n = 2) and ~17% of *Au*. *africanus* (n = 3) have either a fused p4 root or a single root [36]. There is no example of any root fusion (partial or complete) in the p4 of non-hominin fossil apes, and there are only very rare occurrences in *Pan*. In the large tooth samples of extant *Pan* observed in several studies, the hominin condition is present in less than 2–5% [63, 73, 74]. Further, the root configuration in p4 is less variable than in other lower and upper premolars of *Pan* [63]. The inter-genus variability among extant great apes is low, but large between great apes and humans.

**Table 1:** Root and pulp canal configuration in c-m3 of *G*. *freybergi* (holotype, this study) and *O*. *macedoniensis* [32].

	*G*. *freybergi*	*O*. *macedoniensis*	
**c**	1_1_	-	(n = 0)
**p3**	1_1_M+1_2_D	1_1_M+2_2_D	(n = 4)
**p4**	1_1_M+1_2_D	1_2_M+2_2_D	(n = 2)
	(partially fused)	2_2_M+2_2_D	(n = 2)
**m1**	2_2_M+1_1_-_2_D	2_2_M+1_2_D	(n = 4)
**m2**	1-2_2_M+1_1_D	1_2_M+1_2_D	(n = 2)
		2_2_M+1_2_D	(n = 3)
**m3**	1_1_M+1_1_D	1_2_M+1_1_D	(n = 4)
		2_2_M+1_1_D	(n = 1)

The premolars in *G*. *freybergi* have two roots and three pulp canals. The molars are three- or two-rooted and have between four and two pulp canals. M = mesial; D = distal; large cipher = root number; index = pulp canal number; n = sample size for *O*. *macedoniensis*; sample size for *G*. *freybergi* is always n = 1. Formula scheme and detailed root and pulp morphology in Material & Methods and S1 Fig.

**Fig 6:**
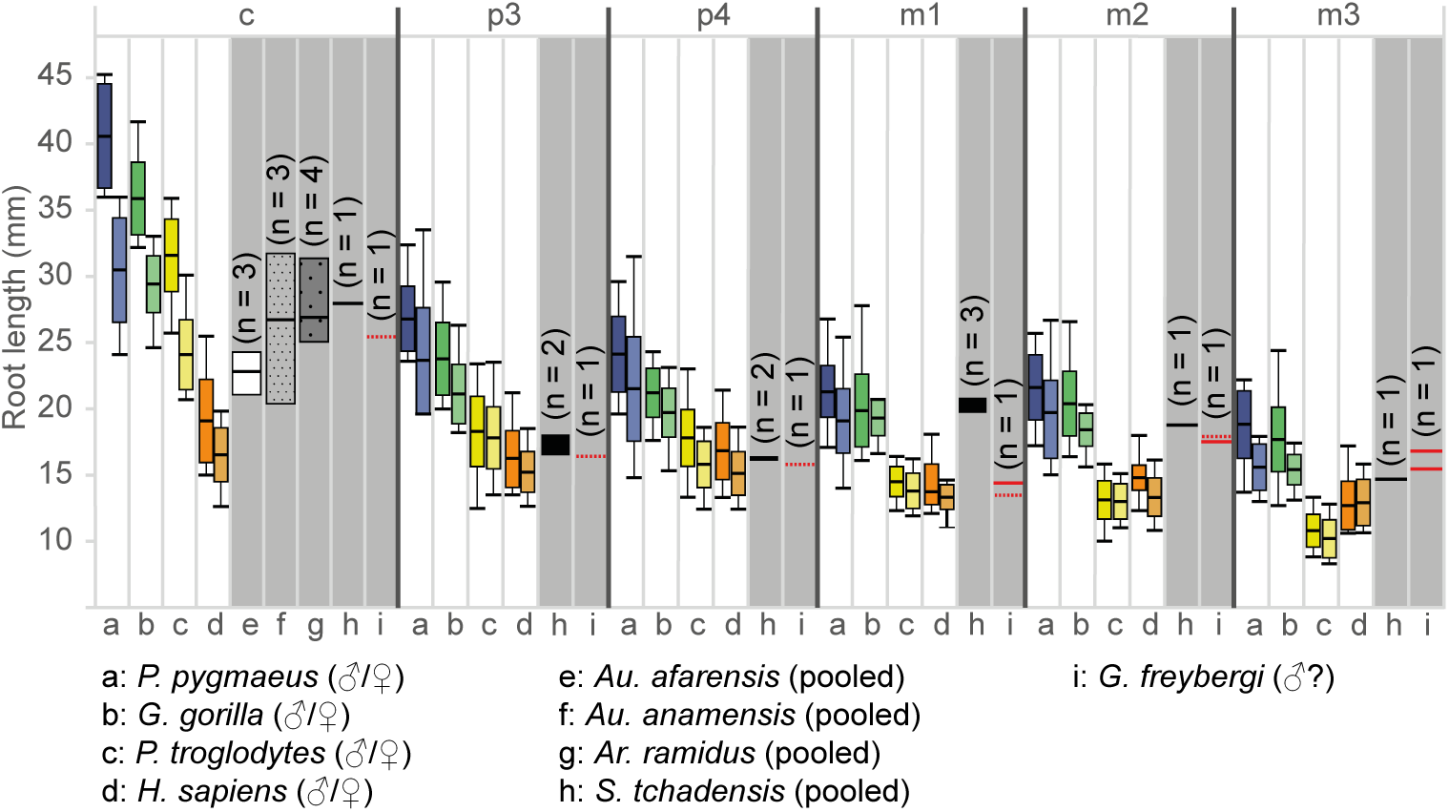
Absolute root lengths of the lower dentition in extant and extinct hominids. The root lengths of extant great apes and humans (min/max/mean/S.D.) are plotted in colour for both sexes (left: males, right: female). The root lengths of fossil taxa (min/max/mean; grey shaded) are sex-pooled. The sample size (n) of fossil taxa is indicated within the graph. For the type of *G*. *freybergi* the root lengths of both hemimandibles are plotted, if preserved. Dashed lines are estimations (see also S3 Fig). Comparative literature data: extant great apes and humans [64]; *Au*. *afarensis* and *Au*. *anamensis* [65]; *Ar*. *ramidus* [31]; *S*. *tchadensis* [28]. Detailed data in S6 Table.

Similar to *O*. *macedoniensis*, the root lengths are rather short compared to extant great apes [32]. In *G*. *freybergi*, this particularly concerns the canine and m1. The absolute canine root length (Fig 6 and S6 Table) is below *S*. *tchadensis* and in the range of *Au*. *anamensis*, *Ar*. *ramidus* and female *P*. *troglodytes*. Given that *G*. *freybergi* may be a male individual, the short canine root may indicate canine reduction. However, this observation needs further confirmation by more canine root length data. The m1 root length is in the range of *P*. *troglodytes* and *H*. *sapiens*, but considerably below *Gorilla* and *S*. *tchadensis*. While in extant great apes and *S*. *tchadensis* the root length of m1 is similar to m2, *G*. *freybergi* shows an m1 root that is considerably shorter than those of m2 and m3.

In *G*. *freybergi*, the radial enamel thickness of the m2 is considerably greater than in extant great apes and *Griphopithecus alpani* (Fig 5 and S5 Table). With l = 1.40 mm it is close to *Griphopithecus darwini* (1.23 mm) and within the mid-range of more thickly enamelled hominins (e.g. *Homo sapiens* l = 1.41 mm). *Ouranopithecus turkae* shows a considerably higher value of l = 2.08 mm. *O*. *macedoniensis* has also a very thick molar enamel [68, 69]. The literature on *O*. *macedoniensis* is not directly comparable to our measurements. However, its relative and absolute molar enamel thickness is reported to exceed that of extant great apes and other Miocene hominids [69].

**Fig 7:**
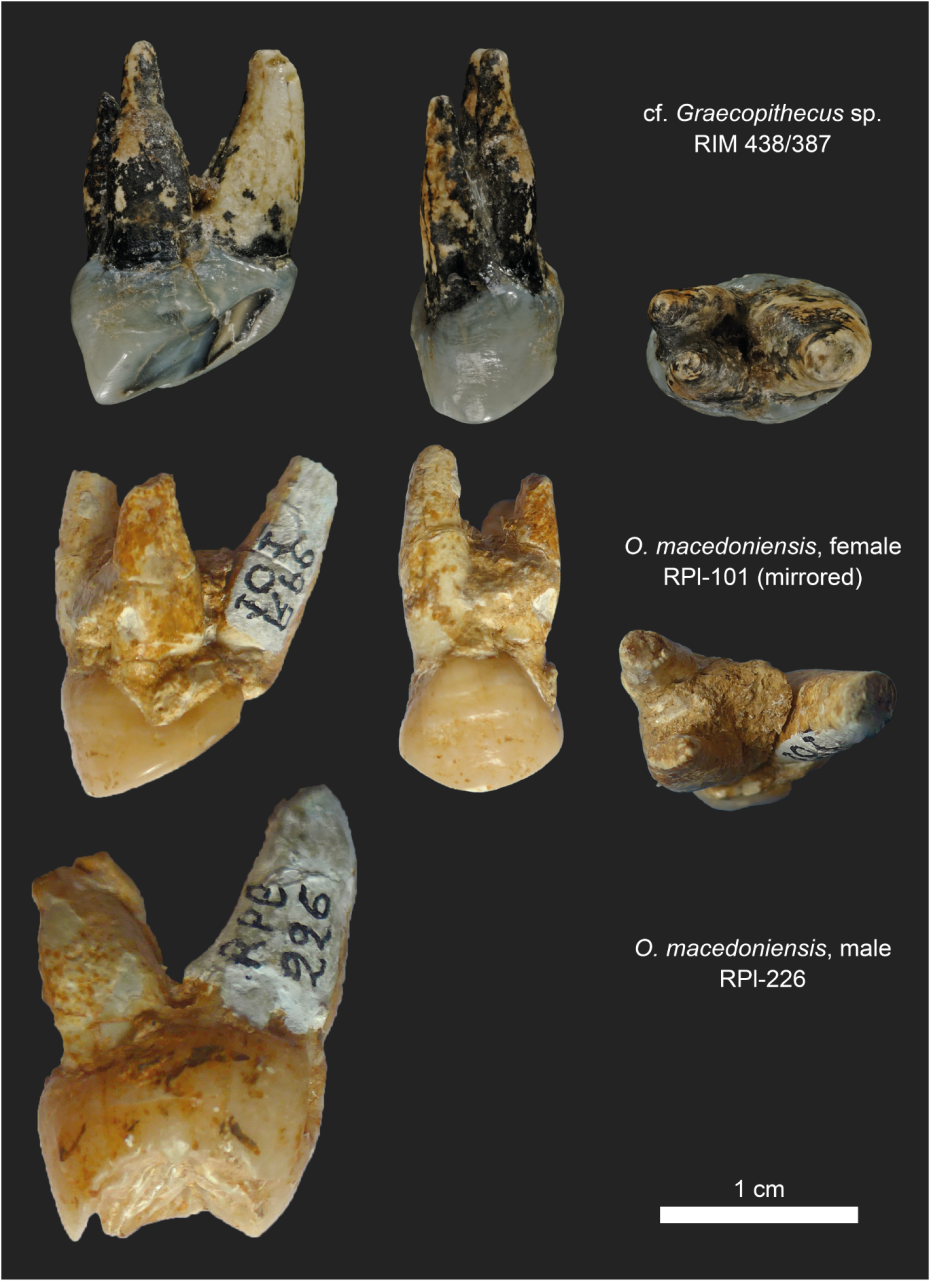
Root morphology in P4 of cf. *Graecopithecus* sp. and *O*. *macedoniensis*. Top row: Left P4 of cf. *Graecopithecus* sp. from Azmaka (Bulgaria) in distal, buccal and apical view. Below: Female and male specimens of *O*. *macedoniensis* from Ravin de la Pluie (Greece). The pictures of the right P4 (RPl-101) are mirrored for a better comparison. (Photos of *O*. *macedoniensis* teeth made with courtesy of G. D. Koufos, Aristotle University of Thessaloniki).

The P4 from Azmaka, Bulgaria is nearly contemporaneous (~65kyr older) with *G*. *freybergi* from Pyrgos [40]. Previously, the P4 had been referred to cf. *Ouranopithecus* sp. or aff. *G*. *freybergi* [39]. This study shows that some morphological aspects are indeed shared with *G*. *freybergi*. The P4 is thickly enamelled, showing the same radial enamel thickness (k = 1.55 mm) as the p4 from Pyrgos (l = 1.50mm). While the size of the Azmaka P4-crown (BL = 12.3 mm; MD = 8.2 mm; Fig 4B) is similar to female *O*. *macedoniensis* (BL = 12.5–13.3 mm; MD = 7.25–9.0 mm), its roots are less robust and more parallel, as in the roots of *G*. *freybergi*. The P4 roots of the female and the larger sized roots of male *O*. *macedoniensis* are more separated and diverge towards the apex (Fig 7). Hence, both individuals from Azmaka and Pyrgos show the same evolutionary trend in upper and lower teeth respectively. Accordingly, we assign the Azmaka specimen to cf. *Graecopithecus* sp.

## Differential diagnosis

*G*. *freybergi* differs from extant great apes (*Pan*, *Gorilla*, *Pongo*) in its thickly-enamelled teeth (Fig 5). It differs from the similar sized *P*. *troglodytes* in its absolutely longer dental roots of m2 and m3, but shows comparable c to m1 root lengths (Fig 6). *G*. *freybergi* differs from most hominids (e.g. *Sivapithecus*, *Ouranopithecus*, australopiths, early *Homo*) in its gracile mandibular corpus (Fig 2). Its corpus height is within the lower range of female *O*. *macedoniensis*, but its breadth is lower. It can be further distinguished from *O*. *macedoniensis* by its narrow dental arc (Fig 3). *G*. *freybergi* differs from *O*. *macedoniensis* in its root configuration, having two-rooted lower premolars including a partially fused p4-root and a reduced number of pulp canals (note the considerations on intra/inter species variation below). It differs from *Ouranopithecus turkae* in having absolutely and relatively thinner enamel and a fused p4-root. The m2 crown size (MD = 14.2mm; BL = 13.2mm) is intermediate between female and male *O*. *turkae*.

## Emended diagnosis

*G*. *freybergi* is a hominid in the size range of female chimpanzees based on dentognathic size. The mandibular dental arch is anteriorly narrow (lingual distance between p3s ≈ 15mm) and diverges slightly posteriorly (lingual distance between m3s ≈ 26mm). The symphysis shows a weak upper and lower transvers torus and a sublingual plane at about 37° relative to the alveolar plane. The mandibular corpus is narrow and deep, which results in a low robusticity index (RI = 0.53 at m2). The posterior dentition is megadont relative to corpus size, with a broad m2 that matches the breadth of the mandibular corpus in this position. Tooth size is estimated to increase from m1 to m3, based mainly on the cervical root area. The enamel is thick (Fig 5 and S5 Table). The dental roots of the tooth row (c to m3) are short (c ≈25.5 mm; p3 ≈16.5 mm; p4 ≈15.9 mm; m1 ≈13.6/ = 14.5 mm; m2 ≈18.0/ = 17.6 mm; m3 = 15.6/16.9 mm; maximum length of left and/or right dentition, derived from μCT based 3D reconstructions, see S3 Fig and S6 Table). The premolars and m3 are two-rooted. The p4 shows a fusion of the mesial and distal root in the upper buccal part. The m1 is three-rooted; the m2 shows three (left) or two (right) roots. Both, m1 and m2 show bifurcated apices in their mesial roots. The molars have low pulp chambers with blunt pulp horns. The number of pulp canals in the postcanine teeth is low (Table 1).

## Phylogenetic position of *Graecopithecus*

The investigation of the internal structures of the Pyrgos mandible reveals characters of the roots of the p4 that are derived compared to other Miocene apes and extant great apes.

In contrast to the Ponginae, *Graecopithecus* shares derived characters with African apes (ventrally shallow roots, buccolingually broad molar roots; [32, 75]). Therefore, we consider four principle alternative interpretations of its phylogenetic position: *Graecopithecus* is a stem-hominine (last common ancestor of African apes and *Homo*), a gorillin, a panin, or a hominin.

**Fig 8:**
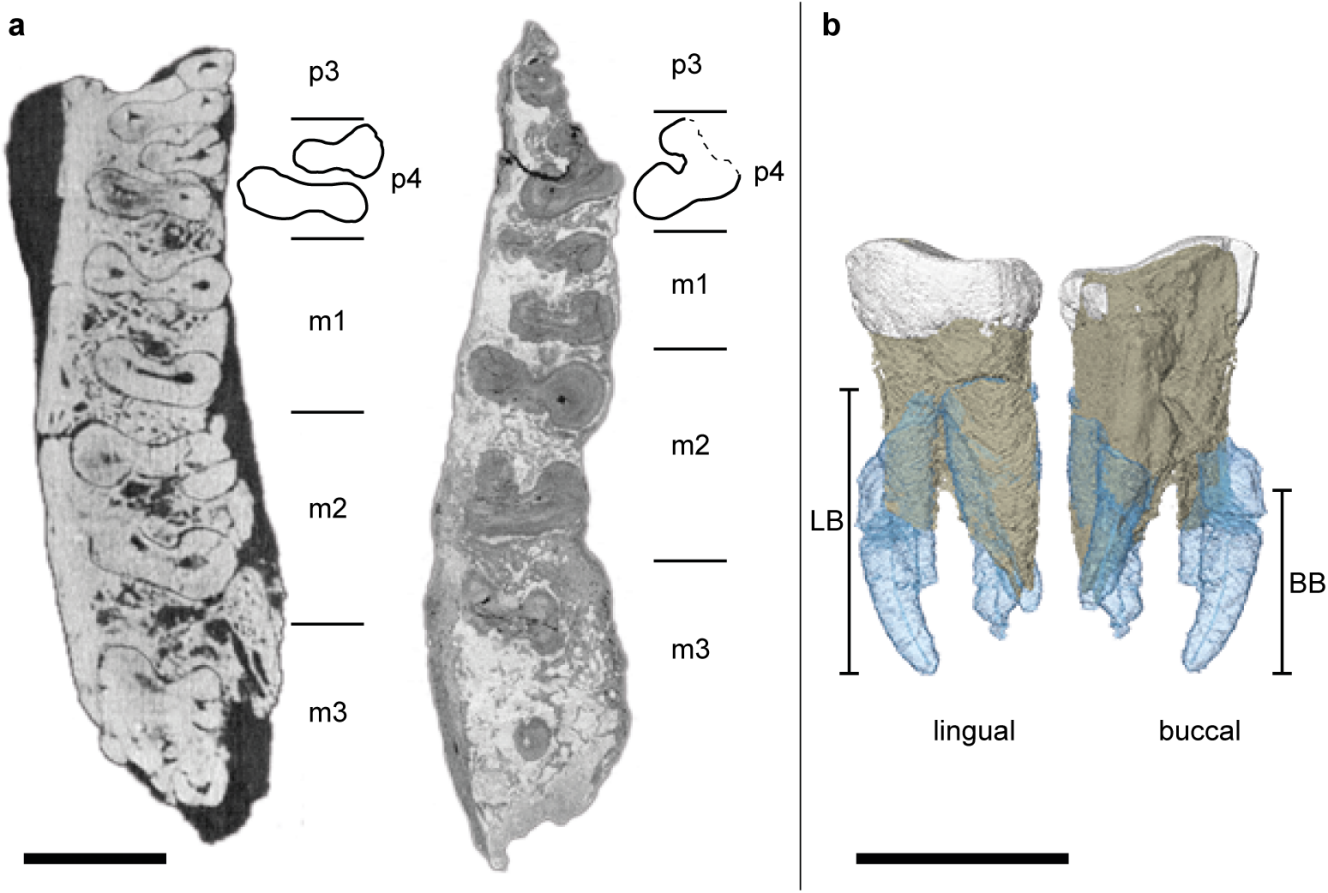
Root morphology of the lower fourth premolar (p4) in *Graecopithecus* and *Sahelanthropus*. **a**, Cervical μCT-section through the right mandibles of *S*. *tchadensis* (left; [29]) and *G*. *freybergi* (right) with drawings of their p4 cross-sections at the level just below the cervix (for *G*. *freybergi* 2.5 mm below p4 cervix). **b**, Root configuration in p4 of *G*. *freybergi*. The apical parts of the right p4 roots are missing, but an approximate reconstruction was done by aligning the mirrored roots of the left p4 (in transparent blue). The left p4 is broken just below the level of bifurcation. LB = height of lingual bifurcation, BB = height of buccal bifurcation (both preserved on the right p4). Scale bar, 10 mm.

Basal hominids like *Proconsul* have two or three clearly diverging roots and four pulp canals (1-2_2_M+1_2_D) in the p4 [28]. The prevailing root configuration in extant great apes is two roots and two to three pulp canals [73], which is the condition seen in *G*. *freybergi* (1_1_M+1_2_D). However, the mesial and the distal roots of *G*. *freybergi* are partially fused at about 47% of maximal root length (Fig 8), a character which is extremely rarely observed in extant great apes (2–4%; [73]). This fusion may represent an early stage of a Tomes’ root, a character that is considered diagnostic for the hominin clade [26, 27]. Thus far, a buccal root fusion similar to *G*. *freybergi* is reported from australopithecines [25, 36]. The configuration of the p4 root and the pulp canal in *G*. *freybergi* is intermediate between the narrow p4 roots in *S*. *tchadensis* [28] (Fig 8) and the Tomes’ root in *Ar*. *kadabba* [76]. The derived state of *G*. *freybergi* with respect to *O*. *macedonensis* is further supported by root and pulp canal reductions in other tooth positions (Table 1). The hominin record shows different levels of p4 root fusion, although separated roots are common as well. However, p4 root fusion never occurs in Miocene non-hominins, suggesting that this feature in *Graecopithecus* is a hominin synapomorphy. Accordingly, the most parsimonious interpretation of the phylogenetic position of *Graecopithecus* is that it is a hominin, although we acknowledge that the known sample of fossil hominin root configurations is too small for definitive conclusions.

A feature supporting this interpretation is the observation of canine root reduction. With an estimate canine root length of ~25.5 mm (Fig 6), the probably male specimen of *G*. *freybergi* is in the range of female *P*. *troglodytes* (24.1 ±2.7 mm [64]) and below female *G*. *gorilla* (29.4 ±2.2 mm). It is in the range of *Au*. *anamensis* (20.3–31.8 mm [65]) and *Ar*. *ramidus* (25.0–31.4 mm [31]). Further, it is shorter than the lower canine root of *S*. *tchadensis* (27.97mm [28]) and above *Au*. *afarensis* (21.0–24.3 mm [65]) and *H*. *sapiens* (16.5±2.1mm [64]).

In earlier studies, a relationship of European hominids to the African hominins is proposed [77, 78]. Taken at face value, the derived characters of *Graecopithecus* (p4 root morphology and possibly canine root length) may indicate the presence of a hominin in the Balkans at 7.2 Ma. In many publications, de Bonis, Koufos and colleagues have proposed that *Ouranopithecus*, from northern Greece and more than 1.5 million years older, is a hominin [47, 79, 80]. Other researchers have interpreted the similarities between *Ouranopithecus* and australopithecines as homoplasies [81]. It is possible that the similarities between *Graecopithecus* and *Ardipithecus* and some australopithecines are also homoplasies. However, as stated before the premolar root number is less functionally constrained than megadonty and enamel thickness, and thus, potentially more useful for phylogeny reconstruction [19, 20]. *Graecopithecus* has reduced root morphology yet heavy mastication and megadontia, suggesting a de-coupling of root and molar function. In contrast, larger roots, large teeth and thicker enamel together contribute to a functional complex shared with australopithecines, which is evoked as the mechanism accounting for the homoplastic appearance of hard object feeding adaptations in *Ouranopithecus* and australopithecines [81].

Therefore, we submit that the dental root attributes of *Graecopithecus* suggest hominin affinities, such that its hominin status cannot be excluded. If this status is confirmed by additional fossil evidence, *Graecopithecus* would be the oldest known hominin and the oldest known crown hominine, as the evidence for the gorillin status of Chororapithecus is much weaker than the hominin status of *Graecopithecus* [8]. More fossils are needed but at this point it seems likely that the Eastern Mediterranean needs to be considered as just as likely a place of hominine diversification and hominin origins as tropical Africa.

## Supporting information

S1 Fig 3D-reconstructions of the P4 from Azmaka (RIM 438/387) and the preserved lower teeth of *G*. *freybergi* from Pyrgos virtually isolated from the type mandible. The P4 is shown in distal and mesial view (top row), and apical and buccal view (bottom row) with associated pulp canals. The lower dentition is shown in distal and mesial view (top row), and apical and lingual view (bottom row) with associated pulp canals. Zoom in for more details. The dashed line indicates the vertical position of the cervical plane constructed as described in Material & Methods and S3 Fig. **a**, Left P4 of cf. *Graecopithecus* sp. and premolars of the right hemimandible of *G*. *freybergi*. **b,** Canine and premolars of the left hemimandible of *G*. *freybergi*. **c,** Molars of the right hemimandible of *G*. *freybergi*. **d,** Molars of the left hemimandible of *G*. *freybergi*.
(TIF)

S2 Fig Micro-CT transverse sections through the left and right mandibular corpus of *G*. *freybergi*. Sections at the level of p4, m1, m2, and m3 (top down), perpendicularly to the alveolar plane. Measurements of mandibular height (H) and breadth (B) in red. The dashed lines indicate surfaces where the cortical bone is crushed or parts of the corpus are missing. Measurements were taken on the better-preserved left corpus. (=) Direct breadth measurements, taken at the positions of m2/m3 and m3. (≥) Minimal estimations after reconstructing minor damages as shown by the dashed line. Minimal estimations are given for the breadth at p3/p4 to m2 and the height at m2, m2/m3 and m3 (S1 Table).
(TIF)

S3 Fig Virtual reconstruction of the Pyrgos mandible with root length measurements and estimated corrections. **a**, Right hemimandible with cervical planes (CP) and root length measurements at the longest radicals of right m1-m3. The CPs are constructed through the cervices of the right m3, m2 and m2-p4. The CP of the right m2-p4 is extended mesially to the position of the missing canine. **b**, In order to define the CPs for the left hemimandible the left tooth row is mirrored (in blue) and aligned to the right tooth row. Thereby, the right CPs are transferred to the left hemimandible. **c**, Mirrored left hemimandible with the root length measurements and estimations at m3-c from the root apices to the constructed CPs.
(TIF)

S1 Table Mandibular corpus dimensions of *G*. *freybergi* and other Miocene and Pliocene hominids. RI = robusticity index. Values in parantheses = corrections for breakage. Data: *G*. *freybergi*: *this study; *Ouranopithecus macedoniensis* (RPl-54: *this study and [47]; RPl-56, 75 and NKT-21: [47]; RPl-89, 90, 80; 94: [48]); *Ankarapithecus meteai*: (AS95-500: [49]); *Sivapithecus sivalensis* and *S*. *punjabicus*: (several samples: [45]); *Nakalipithecus nakayamai*: (KNM-NA46400:[50]); *Australopithecus anamensis* (KNM-KP 29281, 29287, 31713: [51]); *Australopithecus deyiremeda* (BR-VP-3/14; WYT-VP-2/10: [52]); *Australopithecus afarensis*, *Au*. *africanus*, early *Homo*, *Paranthropus robustus and P*. *boisei* (several samples: [52]).
(XLSX)

S2 Table Arcade width in the types of *G*. *freybergi* and *O*. *macedoniensis*. The arcade width at each tooth position was measured at the lingual sides of the dental cervices (lingual distances between the left and right tooth row). The measuring position along the tooth row is given as distance from mid-m3 (average of both sides). All values in mm.
(XLSX)

S3 Table Dental crown dimensions in p4 and m2 of *G*. *freybergi* compared to fossil hominids and chimpanzees. Parantheses indicate estimations. In specimens that preserve the left and right dentition, the mean value of both teeth is given. Data: *G*. *freybergi*: this study; *O*. *macedoniensis*: [47, 48]; *O*. *turkae*: [41]; *N*. *nakayamai*: [50]; *A*. *meteai*: [49, 55]; *S*. *tchadensis*: [3]; *O*. *tugenensis*: [2]; *Ar*. *kadabba*: [9]; *Ar*. *ramidus* and *A*. *afarensis*: [1]; *A*. *anamensis*:[33]; *P*. *troglodytes*: [57].
(XLSX)

S4 Table Dental crown dimensions in P4 of cf. *Graecopithecus* sp., *O*. *macedoniensis* and *O*. *turkae*. In specimens that preserve the left and right dentition, the mean value of both teeth is given. Data: cf. *Graecopithecus* sp.: [39]; *O*. *macedoniensis*: [39, 48, 56]; *O*. *turkae*: [41].
(XLSX)

S5 Table Radial enamel thickness of fossil and extant hominids. Data: cf. *Graecopithecus* sp. and *G*. *freybergi*: this study; *O*. *turkae*: [41]; *Griphopithecus*, *H*. *sapiens* (Ho 08 and Ho23), *P*. *troglodytes*, *G*. *gorilla* and *P*. *pygmaeus*: [59]; *H*. *sapiens* (n = 10): [60]; *H*. *sapiens* (n = 34): [58].
(XLSX)

S6 Table Absolute root lengths in the lower dentition (c-m3) of G. *freybergi* and comparative species. The preserved root length of fragmentary roots is indicated as minimum length (>). Estimations of their maximum length are given for *G*. *freybergi* (in brackets); see also S3 Fig. The measured root positions are indicated as follows: single root (1R), mesial root (m), distal root (d). Data: *G*. *freybergi*: this study; *S*. *tchadensis*: [28]; *Ar*. *ramidus*: [31]; *Au*. *anamensis* and *Au*. *afarensis* [65]; *P*. *pygmaeus*, *G*. *gorilla*, *P*. *troglodytes* and *H*. *sapiens* [64]. For the comparability between studies see in Methods.
(XLSX)

S1 Text Further comparison. (DOCX)
