# Supplementary figures and images for: Potential hominin affinities of Graecopithecus from the Late Miocene of Europe
Source: PLoS One. 2017 May 22;12(5):e0177127. doi: 10.1371/journal.pone.0177127 (PMC5439669; doi:10.1371/journal.pone.0177127)

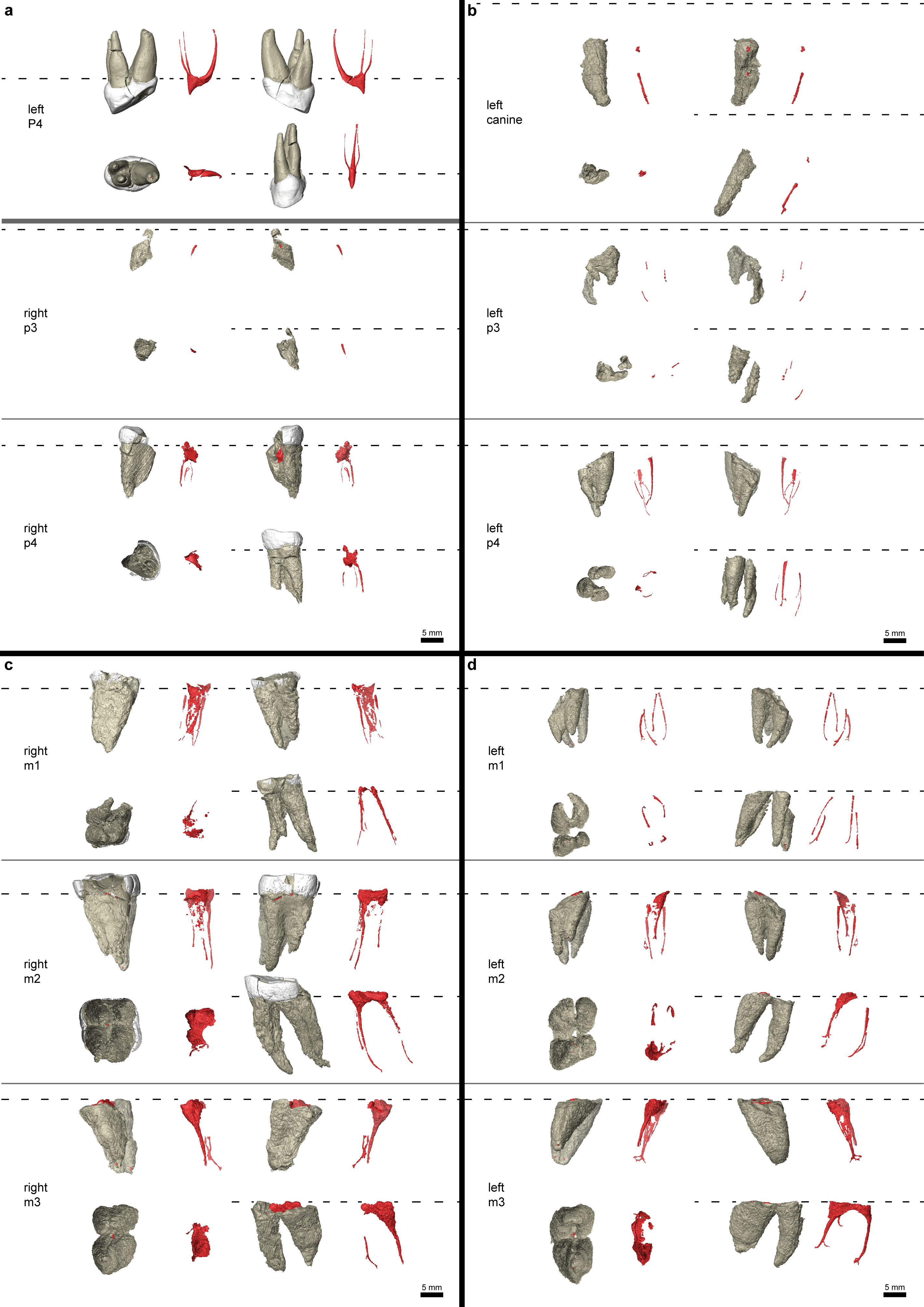

Supplement: S1 Fig — The P4 is shown in distal and mesial view (top row), and apical and buccal view (bottom row) with associated pulp canals. The lower dentition is shown in distal and mesial view (top row), and apical and lingual view (bottom row) with associated pulp canals. Zoom in for more details. The dashed line indicates the vertical position of the cervical plane constructed as described in Material & Methods and S3 Fig. a, Left P4 of cf. Graecopithecus sp. and premolars of the right hemimandible of G. freybergi. b, Canine and premolars of the left hemimandible of G. freybergi. c, Molars of the right hemimandible of G. freybergi. d, Molars of the left hemimandible of G. freybergi. (TIF) [file pone.0177127.s001.tif]

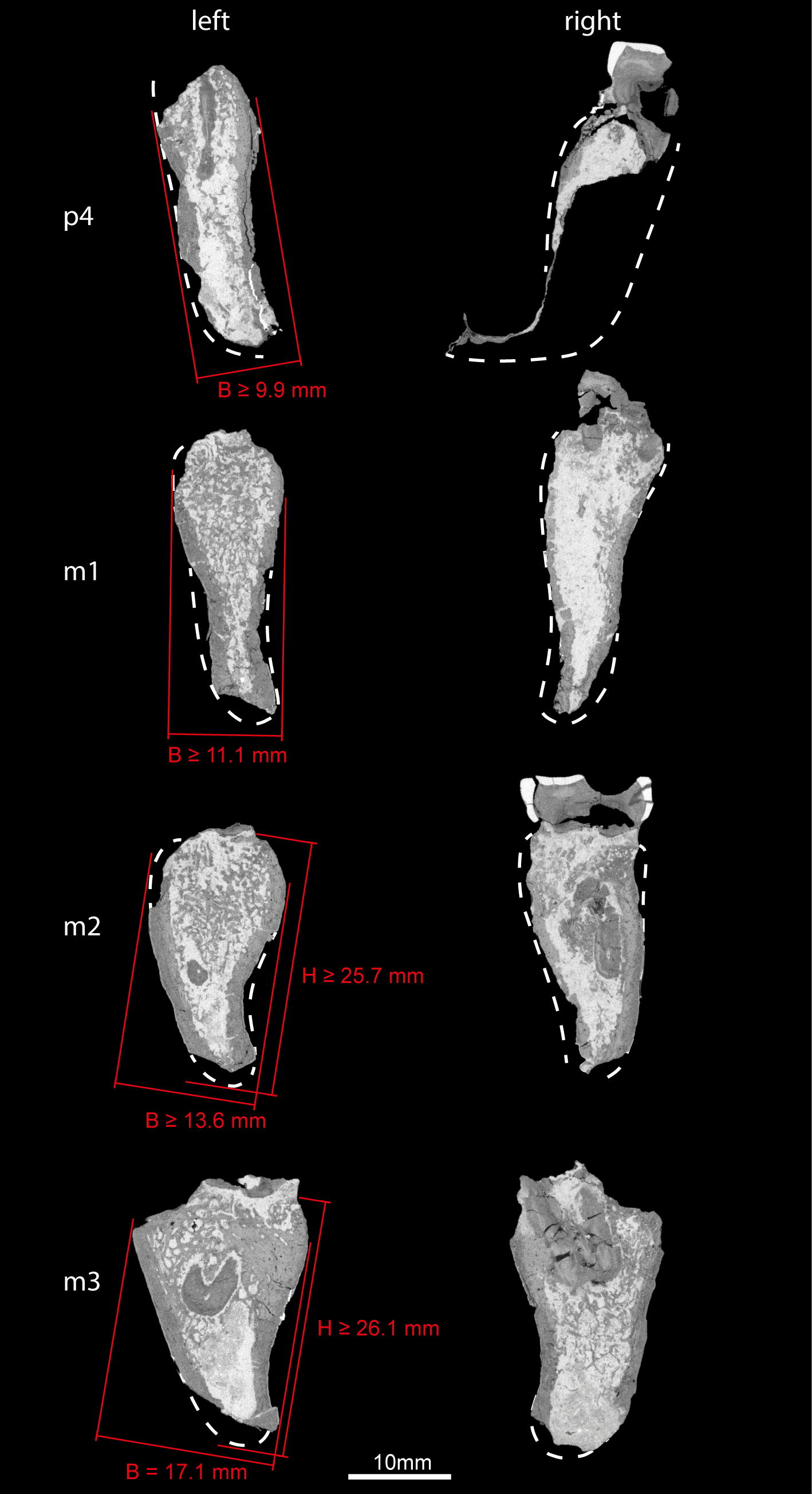

Supplement: S2 Fig — Sections at the level of p4, m1, m2, and m3 (top down), perpendicularly to the alveolar plane. Measurements of mandibular height (H) and breadth (B) in red. The dashed lines indicate surfaces where the cortical bone is crushed or parts of the corpus are missing. Measurements were taken on the better-preserved left corpus. (=) Direct breadth measurements, taken at the positions of m2/m3 and m3. (≥) Minimal estimations after reconstructing minor damages as shown by the dashed line. Minimal estimations are given for the breadth at p3/p4 to m2 and the height at m2, m2/m3 and m3 (S1 Table). (TIF) [file pone.0177127.s002.tif]

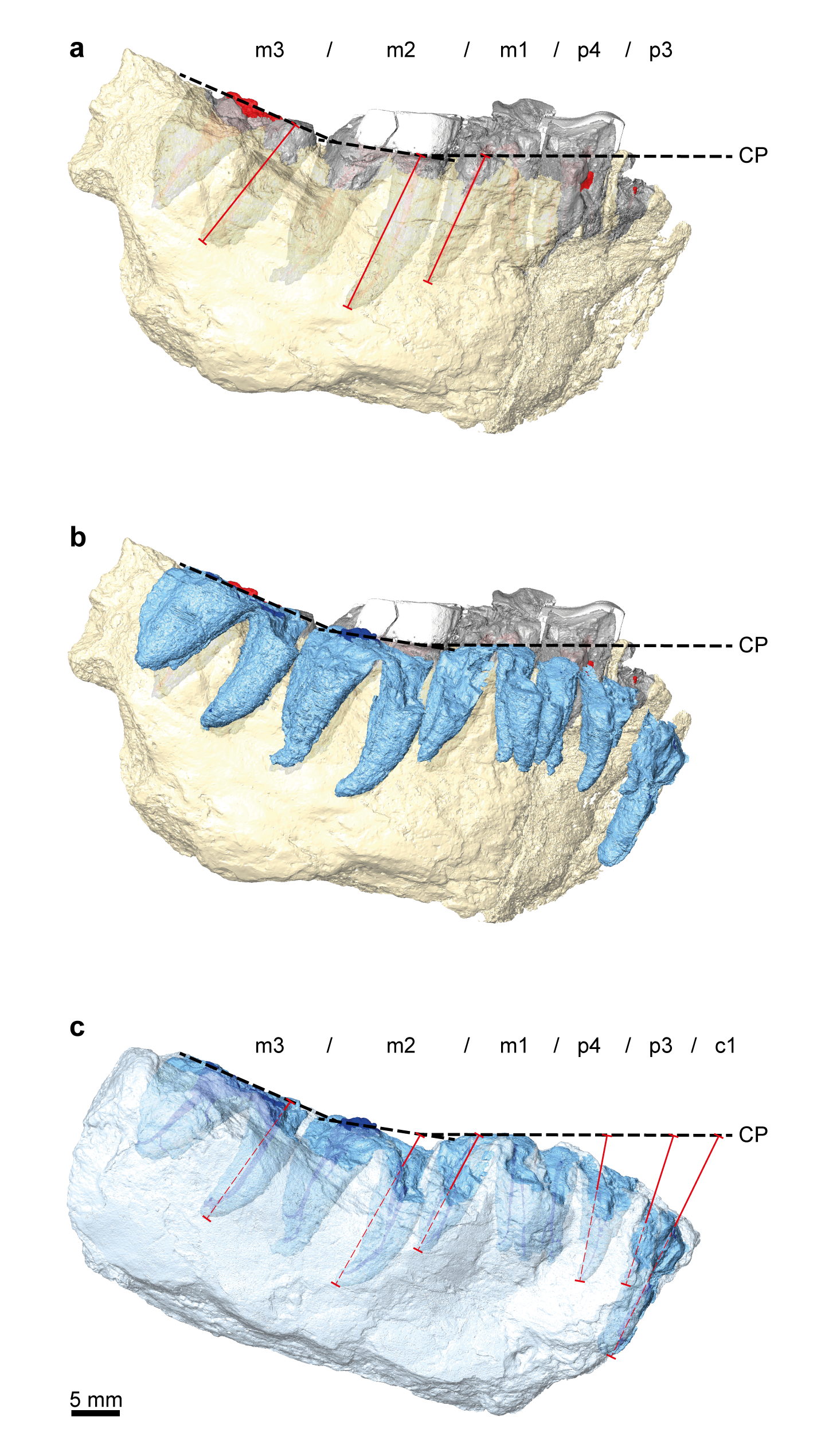

Supplement: S3 Fig — a, Right hemimandible with cervical planes (CP) and root length measurements at the longest radicals of right m1-m3. The CPs are constructed through the cervices of the right m3, m2 and m2-p4. The CP of the right m2-p4 is extended mesially to the position of the missing canine. b, In order to define the CPs for the left hemimandible the left tooth row is mirrored (in blue) and aligned to the right tooth row. Thereby, the right CPs are transferred to the left hemimandible. c, Mirrored left hemimandible with the root length measurements and estimations at m3-c from the root apices to the constructed CPs. (TIF) [file pone.0177127.s003.tif]
