# Supplementary material for: Potential hominin affinities of Graecopithecus from the Late Miocene of Europe
Source: PLoS One. 2017 May 22;12(5):e0177127. doi: 10.1371/journal.pone.0177127 (PMC5439669; doi:10.1371/journal.pone.0177127)
Supplement: S1 Text — (DOCX) [file pone.0177127.s010.docx]

**S1 Text. Further comparison**

In *G. freybergi* the roots of m2 and m3 reach apically deeper into the mandibular corpus than those of m1 and the premolars (Figs 1f-i). A similar pattern is known from *O. macedoniensis*, where all molars roots are positioned apically deeper than the premolar roots [1]. However, this feature is of uncertain usefulness for taxonomy as it might result from wear-related continuous eruption [2]. At least, this seems to be the case for the Pyrgos mandible. The shallow root position of p3-m1 does not solely result from shorter roots. However, the entire tooth in m1 is positioned higher than in m2-m3. With regard to the enormous wear in m1 a continued eruption is evident.

Unlike *O. macedoniensis* and other Miocene to recent great apes the crown of m1 is unusually small relative to m2. This pattern has partially been interpreted as taxonomic relevant, even if interstitial wear has been considered. However, large parts of the dentine are affected by interstitial wear, which alters the cervical outline on mesial and distal face. The distal face is worn up to the distal fossa, losing the complete distobuccal cusp (hypoconulid) and distal marginal ridge. On the mesial face, the m1 lost its complete mesial marginal ridge. Accordingly, a loss of ~30% of its tooth area can be estimated. Thereby, the m1/m2 ratio of *G. freybergi* would be in the range of other hominids [3]. Likewise, the short tooth row of the Pyrgos mandible results largely from closely crowded teeth and a considerable interstitial wear that causes an estimated length reduction of up to ~4 mm in m1. Mesial drifting and high forces during mastication are expected mechanisms leading to these individual features [2].

Shape and angles of the internal symphysis are very similar in *G. freybergi* and *O. macedoniensis* (Fig 3b), contradicting previous descriptions. The t.t.inf. of *G. freybergi* is insignificantly (3mm) more posteriorly expanded than in the type of *O. macedoniensis.* However, these features are within the large variability of other extinct and recent hominids and do not allow a taxonomic assignment on genus level. Hence, significant differences in the symphysal cross-section cannot be confirmed.

1. Emonet EG. Khoratpithecus et la radiation des hominoides en Asie du Sud-Est au Miocène: Université de Poitiers; 2009.

2. Margvelashvili A, Zollikofer CPE, D. L, Peltomäki T, Ponce De Leon MS. Tooth wear and dentoalveolar remodeling are key factors of morphological variation in the Dmanisi mandibles. PNAS. 2013;110(43):17278-83.

3. Evans AR, Daly ES, Catlett KK, Paul KS, King SJ, Skinner MM, et al. A simple rule governs the evolution and development of hominin tooth size. Nature. 2016;530(7591):477-80. doi: 10.1038/nature16972.
